# Target of Rapamycin Coordinates Metabolic Remodeling at the Protein Level in the Red Alga *Cyanidioschyzon merolae*

**DOI:** 10.3390/plants15121790

**Published:** 2026-06-10

**Authors:** Jyothi Priya Putcha, Sousuke Imamura

**Affiliations:** Space Environment and Energy Laboratories, NTT, Inc., Musashino-shi 180-8585, Tokyo, Japan

**Keywords:** microalgae, target of rapamycin, triacylglycerol, starch, red alga, *Cyanidioschyzon merolae*, proteomics

## Abstract

Target of rapamycin (TOR) is a conserved protein kinase that integrates nutrient and energy signals to control growth and metabolism, yet its proteome-level impact in microalgae remains poorly understood. Here, we conducted quantitative proteomics analysis of the unicellular red alga *Cyanidioschyzon merolae* under rapamycin-induced TOR inactivation to characterize global changes in protein abundance. TOR inhibition triggered widespread metabolic remodeling, including coordinated shifts in carbon and nitrogen allocation, and pronounced changes in protein synthesis, photosynthesis, and energy metabolism. Specifically, proteins associated with ribosome biogenesis and ribosomal subunits declined broadly, indicating impaired translation, alongside pronounced reductions in photosynthetic components, including PSI/PSII subunits and chlorophyll biosynthesis enzymes. In contrast, triacylglycerol (TAG) biosynthesis and starch metabolism were enhanced, indicating a shift towards carbon storage. Notably, a diacylglycerol acyltransferase (DGAT; CMQ199C) and a UDP-glucose pyrophosphorylase (UGP; CMS159C) were strongly induced (2.02-fold and 3.48-fold, respectively), identifying them as candidate targets for enhancing TAG and starch accumulation. Proteins associated with nitrogen assimilation were also upregulated, supporting TOR-dependent regulation of nitrogen metabolism at the protein level. Together, these results indicate that TOR orchestrates proteome-level reprogramming in *C. merolae*, coordinating growth, energy production, and carbon storage across interconnected metabolic pathways.

## 1. Introduction

Owing to climate change and the depletion of fossil fuels, global demand for sustainable and renewable energy sources is increasing [1]. Among various alternatives, microalgae have emerged as a promising candidate for biofuel production [2]. As they efficiently convert solar energy, water, and carbon dioxide into valuable biomass [3], microalgae exhibit significantly higher biomass productivity than terrestrial plants; while higher plants typically yield 5–30 tonnes of dry biomass per hectare annually, microalgae can produce 50–150 tonnes [4].

In addition to high productivity, microalgae offer several other advantages, including rapid growth, high photosynthetic efficiency, the ability to accumulate substantial lipid levels, and ease of adaptation to various stresses [5,6,7]. Moreover, microalgae-derived biomass can be produced without competing with agricultural land or food resources [3,6,8]. These features make microalgae attractive cell factories for producing biofuels such as biodiesel and bioethanol [9,10]. The primary energy storage compounds in microalgae—neutral lipids and starch—serve as key raw materials for biofuel production. Neutral lipids are typically stored as cytoplasmic droplets (LDs), mainly in the form of triacylglycerols (TAGs), which are converted into biodiesel [3,11]. Meanwhile, starch, a semi-crystalline glucose polymer, provides a valuable feedstock for producing various industrially relevant bioproducts, such as biofuels, animal feed, and bioplastics [12,13,14].

Current research on microalgal TAGs and starch has gained considerable momentum due to their high potential as biofuel feedstocks [15,16]. In addition to screening for high-yielding strains, researchers are exploring strategies to enhance lipid and starch accumulation by optimizing culture conditions and elucidating underlying metabolic pathways [17,18]. Many studies have reported an increased accumulation of TAG and starch in various microalgae under various stress conditions. For instance, under nitrogen starvation, the eustigmatophyte *Nannochloropsis* amassed neutral lipids of up to 50% of its dry biomass [19]. In contrast, the haptophyte *Isochrysis zhangjiangensis* showed greater accumulation of carbohydrates relative to neutral lipids [20]. Another study using the chlorophyte *Chlamydomonas reinhardtii* reported enhanced TAG and starch levels under separate nitrogen and sulfur starvation [21].

Beyond nutrient stress, recent studies have highlighted the role of the target of rapamycin (TOR) signaling pathway in regulating TAG and starch accumulation in microalgae [22,23,24,25,26,27]. As a conserved eukaryotic serine/threonine kinase, TOR acts as a master regulator of cell growth and metabolism in response to environmental cues, including nutrient availability [28,29,30]. It functions through two distinct complexes: TOR complex 1 (TORC1) and TOR complex 2 (TORC2) [31,32,33]. TORC1, which is well-conserved within diverse eukaryotic lineages, is positively regulated by stimuli such as nutrients, promoting cell proliferation and mass accumulation. TORC2, however, which is absent in plant lineages and remains less well-characterized [34], is primarily responsible for actin organization [35]. Notably, TORC1 is sensitive to inhibition by the drug rapamycin [33,36].

*Cyanidioschyzon merolae* is a primitive, extremophilic red alga that thrives in acid hot springs. It exhibits a simple cellular architecture containing only a single mitochondrion, a single chloroplast, and minimal membranous organelles [37]. Following the sequencing of its complete nuclear and organellar genomes [38,39,40,41], *C. merolae* is characterized by a compact genome with low genetic redundancy. Furthermore, the development of robust molecular tools, including transient gene expression, inducible/repressible transgenes, and homologous recombination, has established this species as a premier model eukaryote [42,43,44,45,46]. Owing to these features, *C. merolae* is widely utilized for studies on the structural and functional characterization of photosynthetic complexes, metabolic pathways, and evolutionary biology.

TOR inhibition in *C. merolae* has emerged as a crucial approach for identifying regulatory mechanisms and underlying genes that control TAG and floridean starch (hereafter referred to as starch) accumulation [22,24,27,47,48,49]. For example, transcriptome analysis has identified GPAT1 as a contributor to increased TAG productivity [48]. Furthermore, phosphoproteomic studies have revealed that the TOR signaling pathway regulates the phosphorylation states of GLG1 (involved in starch synthesis) and GWD (involved in starch degradation). Exploiting this regulatory system has successfully enhanced starch accumulation [24,27]. However, comprehensive knowledge of changes in protein abundance under TOR-inhibited conditions remains limited in *C. merolae* and other algal species.

Therefore, in this study, we performed proteomic profiling of *C. merolae* under rapamycin-induced TOR inhibition. We aimed to elucidate protein level changes, providing fundamental insight into TOR-regulated cellular mechanisms and informing future strategies for enhancing the accumulation of valuable biofuel precursors, such as TAG and starch.

## 2. Materials and Methods

### 2.1. Strain and Culture Conditions

*C. merolae* SF12 strain [47] was cultured in MA2 medium [50] at 40 °C under continuous white light (50 µmol m^−2^s^−1^) with ambient air bubbling. Under the TOR inactivation condition, *C. merolae* SF12 cells (OD_750_ = 0.2–0.4) were exposed to 2 µM rapamycin. In parallel, for the control, the same amount of DMSO (rapamycin solvent) was added to the culture medium [47]. At this dosage (2 µM), rapamycin specifically inhibits TOR activity while causing only minimal growth inhibition, as previously established [24,27,47]. Cell samples from the treatment and control groups, which were run in triplicate, were collected on days 1 and 3 to capture biologically relevant response dynamics. The Day 1 time point reflects early proteomic changes associated with TOR inactivation, while the Day 3 time point represents sustained and compensatory proteomic responses [22,51,52].

### 2.2. Protein Extraction and Data Analysis

Proteins were extracted and processed prior to LC–MS analysis. Briefly, samples were solubilized in a buffer containing 100 mM Tris (pH 8.0), 4% SDS, 20 mM NaCl, and 10% acetonitrile using a sealed ultrasonic homogenizer and rotator. Protein concentration was determined using the bicinchoninic acid (BCA) assay and adjusted to 0.2 μg/μL. SP3 beads and 1-propanol were then added to the protein lysates and mixed for 20 min at room temperature. Subsequently, the beads were washed with 80% 1-propanol followed by ethanol, after which a buffer containing 50 mM Tris-HCl (pH 8.0), 10 mM CaCl_2_, and 0.02% lauryl maltose neopentyl glycol (LMNG) was added and mixed [53]. To fragment protein into peptides, Trypsin/Lys-C mix was added and incubated at 37 °C for 14 h. The resulting peptides were reduced with 10 mM tris(2-carboxyethyl)phosphine (TCEP) and alkylated with 40 mM 2-chloroacetamide at 80 °C for 15 min. Trifluoroacetic acid (TFA) (5%) was then added to the peptides, and desalting was performed using a reverse-phase spin column, after which the peptides were dried using a centrifugal evaporator. Peptides were then reconstituted in 0.1% TFA–0.02% decyl maltose neopentyl glycol (DMNG) to a final concentration of 50 ng/μL with mixing for 10 min. They were then analyzed using liquid chromatography–tandem mass spectrometry (LC–MS/MS) in data-independent acquisition (DIA) mode. NanoLC–MS/MS was performed on an UltiMate 3000 RSLCnano LC system coupled to a Q Exactive HF-X mass spectrometer (Thermo Fisher Scientific Inc., Waltham, MA, USA). Peptides (100 ng) were separated on a 75 μm × 120 mm column (Nikkyo Technos Co., Ltd., Bunkyo-ku, Tokyo, Japan) at 50 °C with a flow rate of 200 nL/min using mobile phases A (0.1% formic acid in water) and B (0.1% formic acid in 80% acetonitrile) under a gradient. The MS was operated in positive electrospray ionization (ESI) mode with DIA acquisition. The total run time was 40 min.

Raw DIA data were processed using DIA-NN (version 1.9.1) [54] with precursor and protein false discovery rates (FDR) controlled at 1%. Protein identification was performed against a reference FASTA database (http://czon.jp/download/ (accessed on 27 January 2025)) [40,41]. Principal component analysis (PCA) was performed using the resulting protein group (.pg) matrix to assess sample variability as a quality-control measure. Downstream data analysis was conducted using Perseus [55]. Protein quantification values were log2-transformed prior to analysis. Proteins quantified in at least 70% of samples in at least one comparison group were retained, while proteins with excessive missing values (i.e., missing values represented as zero intensity) across all groups were excluded. Missing values arising from detection limits reduce data completeness and can affect differential expression analysis [56]. Accordingly, missing values were imputed in Perseus using default parameters (down shift = 1.8, width = 0.3), reflecting the assumption that missing values arise from low-abundance proteins [54]. Using the imputed dataset, fold changes between group means (log2 fold change), *p*-values, and Z-scores were calculated. To correct for multiple testing, permutation-based FDR analysis was performed (250 permutations; FDR < 0.05; S0 = 0.1), and proteins with FDR < 0.05 were considered differentially expressed.

### 2.3. Gene Ontology Biological Process (GO:BP) Enrichment Analysis

Gene ontology biological process (GO:BP) enrichment analysis was carried out in R [57] using the gprofiler2 package (version 0.2.3) [58]. GO:BP enrichment analysis was performed separately for upregulated and downregulated protein groups using g:GOSt tool of gprofiler2 to identify the significant biological processes in each group at each time point (days 1 and 3). Additionally, a custom background consisting of all detected proteins was applied. Statistical significance was assessed using Benjamini–Hochberg FDR correction, with statistical significance set at adjusted *p*-values < 0.05. The top ten GO:BP terms were selected based on the adjusted *p*-value for visualization.

## 3. Results & Discussion

Here, we performed MS-based proteomic profiling of the red alga *C. merolae* under rapamycin-induced TOR inhibition. Quantitative proteome measurements were acquired using a DIA LC–MS/MS workflow, which provides robust and reproducible protein quantification in comparative proteomic analyses [59]. PCA of protein-group-level intensities supported good reproducibility, with consistent clustering of replicates (Figure S1). The reliability of the present dataset is further supported by its concordance with previously characterized TOR-dependent responses in *C. merolae* under comparable experimental conditions, which provide a confident internal reference for evaluating broader trends within the dataset. Notably, the RSH4b protein (CmRSH4b; CMN082C), which has been known to increase in abundance in response to TOR inhibition in *C. merolae* [60], showed a 2.6-fold increase in abundance early after rapamycin treatment, consistent with prior observations. Because RSH4b protein accumulation has been independently quantified under similar experimental conditions [60], its reproducible induction here serves as an internal biological reference supporting the fidelity of the proteomic measurements.

However, we note that changes in protein abundance do not directly establish enzymatic activity or in vivo metabolic outcomes and should therefore not be interpreted as direct functional evidence. Accordingly, the results presented here should be interpreted as correlative rather than mechanistic, and no direct causal relationships between changes in protein abundance and physiological or metabolic outcomes are claimed. In this context, while the specific hypotheses generated from our proteomic resource will require validation through future biochemical, metabolic, and genetics-based functional studies, the present dataset provides a solid foundation for hypothesis generation and a valuable reference framework for the microalgal research community and beyond.

### 3.1. Differentially Expressed Proteins (DEPs) Under Rapamycin Treatment

**Figure 1:**
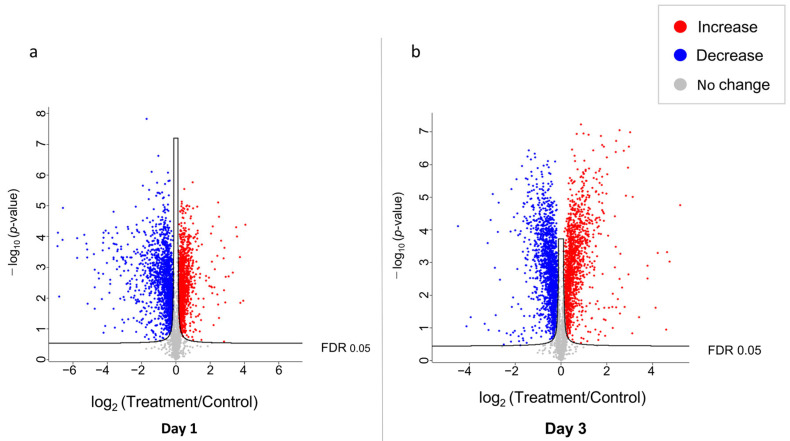
Differentially expressed proteins under control vs. rapamycin treatment in *C. merolae*. Permutation-based false discovery rate (FDR) was performed, and proteins with FDR < 0.05 were denoted as differentially expressed. Volcano plots are generated from the results: red indicates increased expression, blue indicates decreased expression, and grey indicates no significant change at FDR < 0.05. (**a**) Volcano plot showing day 1 detected proteins, where 1244 proteins increased, 1452 proteins decreased, and 1546 proteins were unchanged under rapamycin treatment. (**b**) Volcano plot showing day 3 detected proteins, where 1379 proteins increased, 1635 proteins decreased, and 1190 proteins were unchanged under rapamycin treatment.

We conducted a quantitative proteomic analysis to examine the effects of rapamycin-mediated TOR inhibition on the *C. merolae* proteome. After data processing and quality filtering, 2696 proteins on day 1 and 3014 proteins on day 3 were identified as differentially expressed at FDR < 0.05. On day 1, 1244 DEPs were upregulated, and 1452 were downregulated (Figure 1a). By day 3, 1379 upregulated and 1635 downregulated DEPs were observed (Figure 1b).

**Figure 2:**
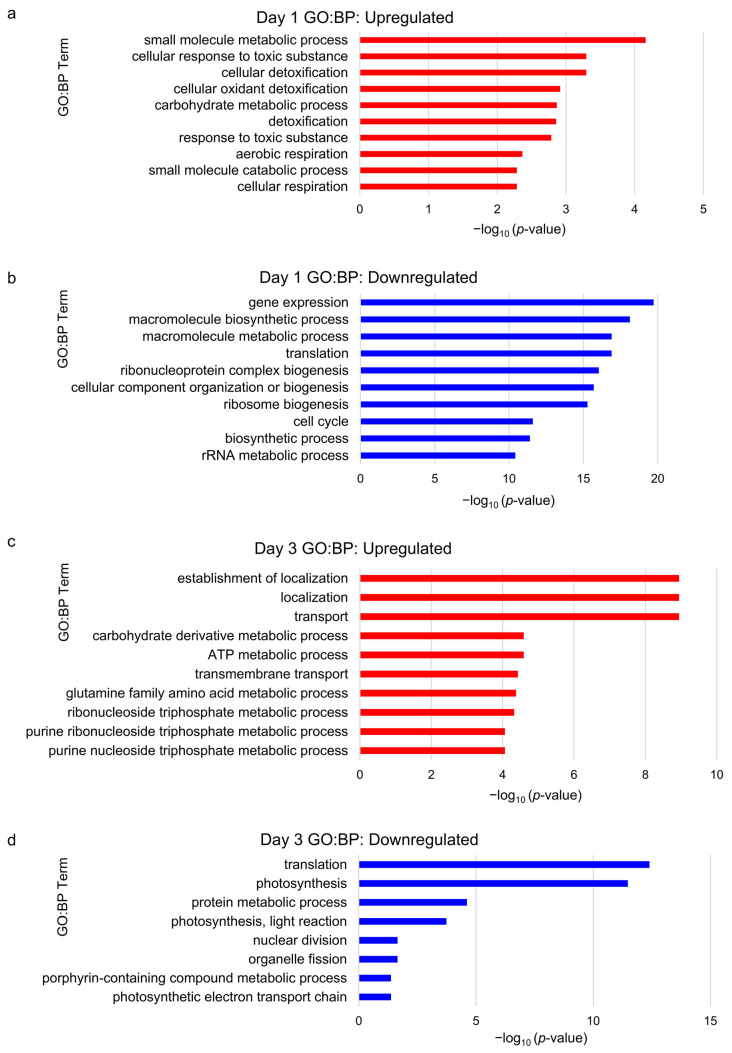
Top 10 significant biological processes from GO:BP enrichment analysis of days 1 and 3 increased and decreased proteins under rapamycin treatment. Colors indicate the protein expression levels: red is increased; blue is decreased. (**a**) Day 1 GO:BP: Upregulated; (**b**) Day 1 GO:BP: Downregulated; (**c**) Day 3 GO:BP: Upregulated; (**d**) Day 3 GO:BP: Downregulated.

We performed a GO:BP enrichment analysis for these upregulated and downregulated DEPs to identify the biological processes significantly impacted by TOR inactivation (Figure 2).

Within day 1 of TOR inhibition, we observed an upregulation of proteins involved in small-molecule metabolic processes, cellular responses to toxic substances, carbohydrate metabolic processes, and cellular respiration (Figure 2a). By day 3, key upregulated processes included transport and localization, carbohydrate derivative metabolic process, ATP metabolic process (purine ribonucleoside triphosphate metabolic process), and glutamine family amino acid metabolic process (Figure 2c). As TOR is a master regulator of nutrient and energy signaling, its inhibition significantly redirects carbon, nitrogen, and energy intermediates within the algal cell [23,34]. Previous studies highlight the carbon and nitrogen allocation, as observed in increases in carbon reserves such as starch and TAGs [22,24] and in amino acids such as glutamate and glutamine after TOR inhibition [23,61]. Ref. [62] reported that metabolic functions associated with transport were among those showing the largest flux changes in *C. reinhardtii* under rapamycin treatment. Thus, cellular transport is also affected, owing to the redirection of various nutrient and energy molecules. Consistent with these previous findings, our observations revealed increased dynamic redistribution of various small molecules—including sugars and amino acids—across cellular compartments in *C. merolae*. This may reflect broader metabolic remodeling under TOR inhibition, potentially involving coordination of carbon and nitrogen allocation and increased carbon flow toward storage reserves.

As for the downregulated processes under TOR inactivation, on day 1 we observed decreases in protein-synthesis-related and cell cycle processes (Figure 2b). TOR signaling directly controls cell growth while indirectly affecting the cell cycle through its regulation of cellular growth and anabolic processes [29,63]. We observed a decrease in translation, ribosome biogenesis, and rRNA metabolic processes. This observation is consistent with previous findings in *C. merolae*, where polysome formation decreased after TOR inactivation [64]. Furthermore, inhibition of protein synthesis in *C. reinhardtii* and other algae has also been reported under rapamycin treatment [34,65]. These findings suggest that TOR signaling regulates protein synthesis in *C. merolae*, as in other eukaryotes, highlighting its functional conservation.

In addition to a decline in translation-related proteins, photosynthesis was majorly downregulated on day 3 (Figure 2d). Additionally, we observed a decrease in porphyrin-containing compound metabolic processes, which is essential for chlorophyll biosynthesis. Similar trends have been reported in other microalgae, including *Nannochloropsis* [66] and *Auxenochlorella* [67], wherein TOR inhibition led to reduced gene expression involved in photosynthesis and chlorophyll biosynthesis. Beyond microalgae, TOR has been implicated in regulating photosynthesis in higher plants [68,69]. For instance, in rice, TOR inhibition resulted in defective thylakoid grana architecture, indicating impaired photosynthetic capacity [70]. Taken together, although the role of TOR signaling in chloroplast development, chlorophyll biosynthesis, and the overall photosynthetic apparatus is yet to be fully understood, the overall suppressive effect of TOR inhibition on photosynthesis appears to be widely conserved across photosynthetic eukaryotes.

### 3.2. TAG Biosynthesis-Related Proteins Increased by TOR Inactivation in C. merolae

**Figure 3:**
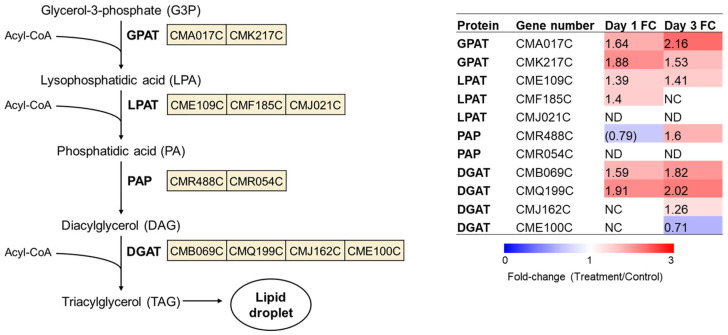
Expression levels of proteins involved in the TAG biosynthesis pathway under rapamycin treatment on days 1 and 3 in *C. merolae*. The gene information is based on [22]. The table on the right shows the proteins along with their fold-change (FC) values on days 1 and 3, with the colors denoting the degree of fold-change: red denotes an increase in the protein level, blue a decrease. Proteins that show no significant change at FDR < 0.05 cutoff and those not detected are denoted as NC and ND, respectively. Proteins for which the FC values (in parentheses) were deemed significant after FDR correction (FDR < 0.05) had corresponding raw *p*-values greater than 0.05 (GPAT: glycerol-3-phosphate acyltransferase; LPAT: lysophosphatidic acid acyltransferase; PAP: phosphatidic acid phosphatase; DGAT: diacylglycerol acyltransferase).

To determine how TOR inactivation influences carbon-storage-related metabolic pathways, we first examined changes in proteins involved in TAG biosynthesis. Rapamycin treatment led to a general increase in TAG biosynthesis-related proteins on both days 1 and 3 (Figure 3).

By day 3, proteins corresponding to each enzymatic step of the TAG biosynthetic pathway were elevated, including GPAT (CMA017C, CMK217C), LPAT (CME109C), PAP (CMR488C), and DGAT (CMB069C, CMQ199C, CMJ162C). Notably, CMA017C (GPAT) and CMQ199C (DGAT) showed more than a two-fold increase.

Previous transcriptomic analyses in *C. merolae* reported increased expression of GPAT genes (*CMA017C*, *CMK217C*) and the DGAT gene (*CMB069C*) under TOR inhibition [22]. *CMA017C* encodes an ER-localized GPAT that functions as a rate-limiting enzyme, and its overexpression results in a 56.1-fold increase in TAG productivity [48]. In contrast, overexpression of *CMB069C*, a type-2 DGAT, did not enhance TAG accumulation. Although DGATs are widely recognized as rate-limiting enzymes in microalgal TAG biosynthesis [71,72,73], the specific DGAT responsible in *C. merolae* has remained unclear. Our proteomic data identify CMQ199C as a strongly induced DGAT (2.02-fold on day 3). The LPAT-catalyzed step has also been proposed as rate-limiting in TAG synthesis. Although the previously characterized LPAT, CMJ021C [49], was not detected in our dataset, another LPAT, CME109C, was upregulated. Additionally, the PAP protein CMR488C increased by 1.6-fold. These enzymes upregulated under TOR inhibition may therefore represent promising targets for improving TAG productivity and warrant further investigation in future studies.

**Figure 4:**
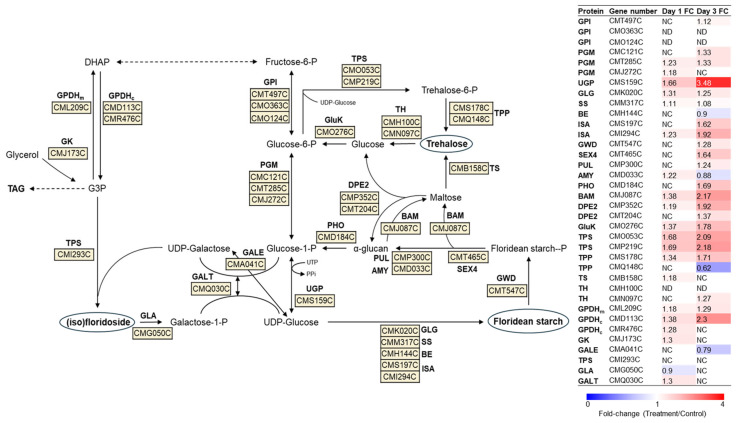
Expression levels of proteins involved in the storage sugar metabolic pathway under rapamycin treatment on days 1 and 3 in *C. merolae*. Gene annotations are based on [24,79,80,81,82]. The table on the right shows the proteins along with their fold-change (FC) values on days 1 and 3. Colors indicate the magnitude and direction of fold-change: red denotes an increase in the protein level, blue a decrease. Proteins that show no significant change at FDR < 0.05 cutoff and those not detected are denoted as NC and ND, respectively (DHAP: dihydroxyacetone phosphate; Fructose-6-P: fructose-6-phosphate; GPDHc: cytoplasmic glycerol-3-phosphate dehydrogenase; GPDHm: mitochondrial glycerol-3-phosphate dehydrogenase; GK: Glycerol kinase; GPI: glucose-6-phosphate isomerase; Glucose-6-P: glucose-6-phosphate; PGM: phosphoglucomutase; Glucose-1-P: glucose-1-phosphate; UGP: UDP-glucose pyrophosphorylase; UTP: uridine-5’-triphosphate; GLG: glycogenin; SS: starch/glycogen synthase; BE: branching enzyme; ISA: isoamylase; GWD: glucan water dikinase; SEX4: dual-specificity phosphatase starch excess 4; BAM: β-amylase; PUL: pullulanase; AMY: α-amylase; DPE2: 4-α-glucanotransferase; PHO: phosphorylase; GALE: UDP-glucose-4-epimerase; G3P: glycerol-3-phosphate; Trehalose-6-P: trehalose-6-phosphate; TPS: trehalose-6-phosphate synthase; TPP: trehalose-6-phosphate phosphatase; TS: trehalose synthase; TH: trehalase; GluK: glucokinase; GLA: α-galactosidase; Galactose-1-P: galactose-1-phosphate; GALT: galactose-1-phosphate uridylyltransferase).

G3P serves as the immediate substrate for TAG biosynthesis (Figure 3), and under TOR-inactivation conditions, protein content of GPDHc, which catalyzes the reduction of DHAP to G3P, was increased by 2.3-fold (Figure 4). This suggests increased protein abundance of enzymes linked to G3P production, which may be associated with conditions favorable for TAG accumulation.

TAG synthesis requires acyl-CoA, prompting us to examine fatty acid biosynthesis and desaturation. Proteins involved in fatty acid synthesis were generally decreased after rapamycin treatment (Table S1), consistent with earlier transcriptomic data in *C. merolae* [22] and observations in other microalgae under nitrogen depletion [74,75]. Although this result appears counterintuitive because fatty acids are required for TAG synthesis, it may reflect reduced membrane lipid synthesis following growth arrest, which would allow newly synthesized fatty acids to be redirected toward TAG production [22].

In contrast, fatty acid desaturation showed a distinct response. The Δ9 desaturase CMJ201C was strongly upregulated (5.98-fold on day 3). This enzyme introduces a double bond at the Δ9 position of stearoyl-CoA to produce oleoyl-CoA (C18:1Δ9-CoA) [76,77]. Consistent with this, rapamycin treatment increases the proportion of 18:1 fatty acids in TAG [22]. Thus, the induction of CMJ201C may be associated with previous findings showing an increased proportion of 18:1 fatty acids in TAG under TOR inhibition. In contrast, nitrogen depletion leads to TAG enriched in 18:2 fatty acids [22,78], indicating that TOR inhibition and nitrogen depletion, while both promoting TAG accumulation, impose distinct metabolic states on lipid biosynthesis.

### 3.3. Changes in Storage Sugar Metabolism-Related Proteins Under TOR Inactivation in C. merolae

#### 3.3.1. Floridean Starch Metabolism Under TOR Inactivation in *C. merolae*

Subsequently, we quantified protein levels associated with starch metabolism—such as TAG, a carbon storage molecule—which accumulates under TOR inhibition. Overall, proteins associated with carbohydrate metabolism exhibited a general upregulation on both days 1 and 3, with expression levels further increasing from day 1 to day 3 (Figure 4).

Under TOR inactivation by rapamycin, the protein content of enzymes involved in starch biosynthesis, such as GPI, PGM, UGP, GLG, SS, and ISA, was generally increased by day 3. Previous studies have identified several starch-biosynthesis-related enzymes as critical drivers of starch accumulation. For instance, Ref. [83] reported that PGM mediates a key rate-limiting step in starch synthesis and contributes to high levels of starch in *Chlamydomonas*. Ref. [24] reported that overexpression of *CMK020C*, encoding GLG, in *C. merolae* resulted in a 4.7-fold increase in starch content. In this study, we identified the protein UGP, encoded by *CMS159C*, which showed a 3.48-fold increase in its levels on day 3. UGP catalyzes the synthesis of UDP-glucose, which is widely regarded as a key upstream step supplying UDP-glucose for starch biosynthesis. In the diatom *Phaeodactylum tricornutum*, UGP enzyme overexpression enhanced the accumulation of chrysolaminarin, the principal carbohydrate storage polysaccharide [84], whereas UGP silencing reduced chrysolaminarin content and promoted lipid accumulation [85]. These findings suggest that UGP is associated with carbon allocation between carbohydrate and lipid storage pathways. Therefore, UGP represents a candidate protein for future functional studies aimed at understanding carbon allocation. Although functional characterization of UGP in red algae remains limited, our results highlight the importance of further investigating UGP and other carbohydrate-metabolism-related proteins identified as upregulated under TOR inactivation in *C. merolae*.

In addition to increased levels of starch biosynthetic enzymes, proteins involved in starch degradation, such as GWD, SEX4, BAM, PUL, PHO, and DPE2, also showed increased levels under TOR-inactivation conditions. This simultaneous upregulation of both synthesis and degradation pathways suggests the potential for dynamic starch turnover, which may reflect active remodeling of carbohydrate metabolism in response to TOR inactivation in *C. merolae*. However, direct evidence of flux changes would require complementary metabolomic or functional analyses.

#### 3.3.2. Preferential Accumulation of Trehalose over (Iso)floridoside Under TOR Inactivation in *C. merolae*

Beyond starch metabolism, we next examined other carbohydrate-related pathways that may also contribute to carbon allocation under TOR inactivation. (Iso)floridoside metabolism-related protein levels showed no significant change following TOR inactivation at the FDR < 0.05 cutoff across the time course, whereas trehalose-metabolism-related proteins showed an increase (Figure 4). Floridoside, (iso)floridoside, and trehalose function as osmolytes and are often accumulated at high levels in response to stress conditions [86,87,88,89]. Among the proteins associated with trehalose metabolism, the TPS proteins CMO053C and CMP219C showed 2.09- and 2.18-fold increases, respectively, on day 3. TPS catalyzes the formation of trehalose-6-P from UDP-glucose and glucose-6-P. Trehalose-6-P in itself is a key signaling metabolite that reflects cellular sugar status and plays an important role in regulating carbohydrate utilization in plants [90,91]. While many red algae harbor highly conserved genes homologous to plant trehalose biosynthetic genes, trehalose itself was long considered rare or absent due to limited and ambiguous detection [92]. However, recent evidence confirms its presence in multiple species, including *Corallina officinalis*, *Cystoclonium purpureum*, *Ahnfeltia plicata*, *Euthora cristata*, and *Porphyra umbilicalis* [93,94]. The increased levels of trehalose-synthesis-related proteins here suggest that *C. merolae* is likely to produce trehalose conditionally, which may function as a temporary carbon reserve or as a signaling molecule in the form of trehalose-6-P. However, these possibilities remain speculative at present and will require direct experimental validation, including metabolite quantification and functional analyses.

Overall, proteins associated with TAG biosynthesis, starch, and trehalose metabolism were upregulated, with expression levels increasing over time, as observed on day 3. This pattern suggests a more sustained and coordinated metabolic reprogramming of carbon allocation in response to TOR inhibition.

### 3.4. Nitrogen-Assimilation-Related Protein Levels Under TOR Inactivation Conditions

In *C. merolae*, TOR inhibition by rapamycin increases the transcript levels of genes involved in nitrogen assimilation, suggesting that TOR signaling plays a regulatory role in nitrogen assimilation [22]. Therefore, we investigated how nitrogen-assimilation-related proteins [95] changed under rapamycin-induced TOR inactivation conditions. The protein abundances of nitrite reductase (CmNiR, CMG021C), glutamine synthetase (CmGS, CMI233C), ammonium transporter (CmAMT1, CMT526C), and glutamate synthase (CmGOGAT, CMV060C) increased by approximately 36.72-fold, 1.59-fold, 2.72-fold, and 1.16-fold, respectively, on day 3 (Table S2). In contrast, the abundance of CmMYB1 (CMJ282C) decreased by approximately 6.3-fold on day 3 (Table S2). Notably, the proteins CmNRT (CMG018C), CmNR (CMG019C), and CmAMT2 (CMK126C) were either not detected or showed no significant changes in abundance. CmMYB1 is a transcription factor that positively regulates the expression of nitrogen assimilation genes, and its mRNA and protein levels are known to increase prior to the induction of nitrogen assimilation genes [44]. Moreover, the magnitude of CmMYB1 mRNA induction upon TOR inhibition is clearly lower than that of nitrogen assimilation genes [22]. Based on these previous observations, the decrease in CmMYB1 observed on day 3 following TOR inhibition in this study may reflect this regulatory behavior. Overall, the results of our proteome analysis are fully consistent with prior transcript-level studies and strongly support the conclusion that the TOR signaling pathway regulates nitrogen assimilation not only at the mRNA level but also at the protein level in *C. merolae*.

### 3.5. Protein-Synthesis-Related Protein Expression Under TOR Inactivation in C. merolae

GO:BP analysis showed that protein-synthesis-related biological processes were significantly affected by TOR inactivation as early as day 1 (Figure 2b), suggesting a rapid cellular response that remained sustained through day 3 (Figure 2d).

**Figure 5:**
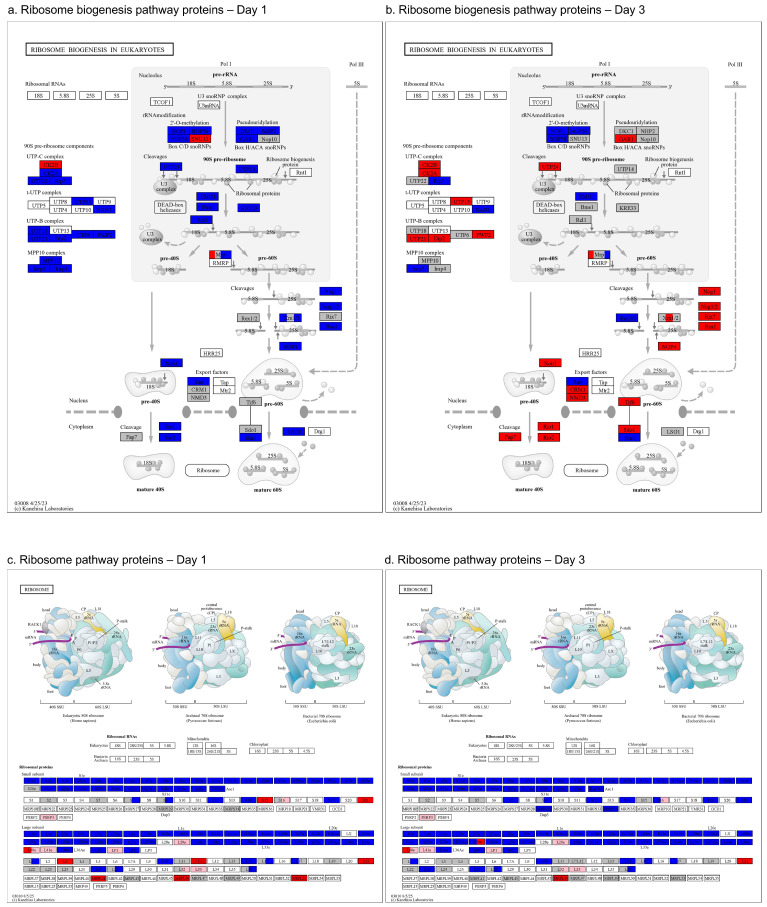
Expression levels of proteins involved in ribosome biogenesis and ribosome under rapamycin treatment on days 1 and 3 in *C. merolae*. The gene information is based on KEGG Pathway maps cme03008 and cme03010 obtained via a KEGG mirror site under a commercial-use license [96]. (**a**) Ribosome biogenesis proteins—Day 1. The expression levels of the associated proteins under rapamycin treatment from day 1 are shown here. (**b**) Ribosome biogenesis proteins—Day 3. The expression levels of the associated proteins under rapamycin treatment from day 3 are shown here. (**c**) Ribosome proteins—Day 1. The expression levels of the associated proteins under rapamycin treatment from day 1 are shown here. (**d**) Ribosome proteins—Day 3. The expression levels of the associated proteins under rapamycin treatment from day 3 are shown here. Colors indicate the protein expression levels: red indicates increased, blue indicates decreased, grey indicates no significant change at FDR < 0.05, and pink indicates those not detected. The figures were generated using the Color tool of KEGG Mapper at a KEGG mirror site under a commercial-use license.

Therefore, we examined the abundance of proteins involved in protein synthesis, mainly those associated with the pathway maps: ribosome biogenesis (KEGG pathway map identifier: cme03008) and Ribosome (KEGG pathway map identifier: cme03010) from KEGG (Kyoto Encyclopedia of Genes and Genomes) [96]. The majority of the proteins involved in ribosome biogenesis decreased on day 1 (Figure 5a, Table S3). By day 3, in contrast to the initial decrease, we observed a partial reversal in protein abundance, with several ribosome-biogenesis-associated proteins increasing after their initial decrease on day 1 (Figure 5b, Table S3). Although the basis of this increase cannot be confirmed, we may assume that ribosome biogenesis, being a highly modular and adaptive process, enters a new steady state as compensatory feedback while remaining under inhibition. The compensatory feedback may be observed in the modest rebound of the few ribosome biogenesis proteins (Figure 5b, Table S3), including the RNA polymerase proteins such as CMS114C and CMT451C (Table S4). It has been speculated that transcriptional regulation of specific ribosomal proteins might contribute to proteostasis recovery [97].

As for the ribosome proteins (cme03010) (r-proteins), we observed that on both days 1 and 3, the majority were decreased and remained at similarly low levels (Figure 5c,d; Table S5). In *Saccharomyces cerevisiae*, TOR inactivation sharply reduces the production of newly synthesized ribosomal subunits in parallel with a marked decline in r-protein production [98], and TOR kinase not only regulates translational initiation but is also required for the continued transcription of r-protein genes [99,100]. A similar reduction in rRNA and ribosomal protein synthesis was observed in *Arabidopsis* [101]. Reduced TOR activity has been linked to reduced protein synthesis in microalgae [64,65]. Together, these studies support a multi-layer model in which TOR controls protein synthesis at several levels: transcription of r-protein genes, rRNA production/processing, and translational initiation. The protein abundance profiles here suggest a coordinated downregulation of the transcription and translation of mRNAs coding for ribosomal proteins, leading to a decline in the r-proteins, thereby affecting translation. The general involvement of TOR in protein synthesis is therefore a widely conserved response in eukaryotes; however, the exact mechanisms of this regulation in this alga require further investigation.

### 3.6. Expression of Photosynthesis-Related Proteins Under TOR Inactivation in C. merolae

In photosynthetic eukaryotes, photosynthesis is essential for biomass production, and its efficiency represents a major limiting factor for productivity. Although TOR signaling has been implicated in regulating photosynthesis, the underlying mechanisms remain poorly understood. Therefore, identifying proteins affected by TOR inactivation may help shed light on how TOR influences photosynthetic growth [102].

**Figure 6:**
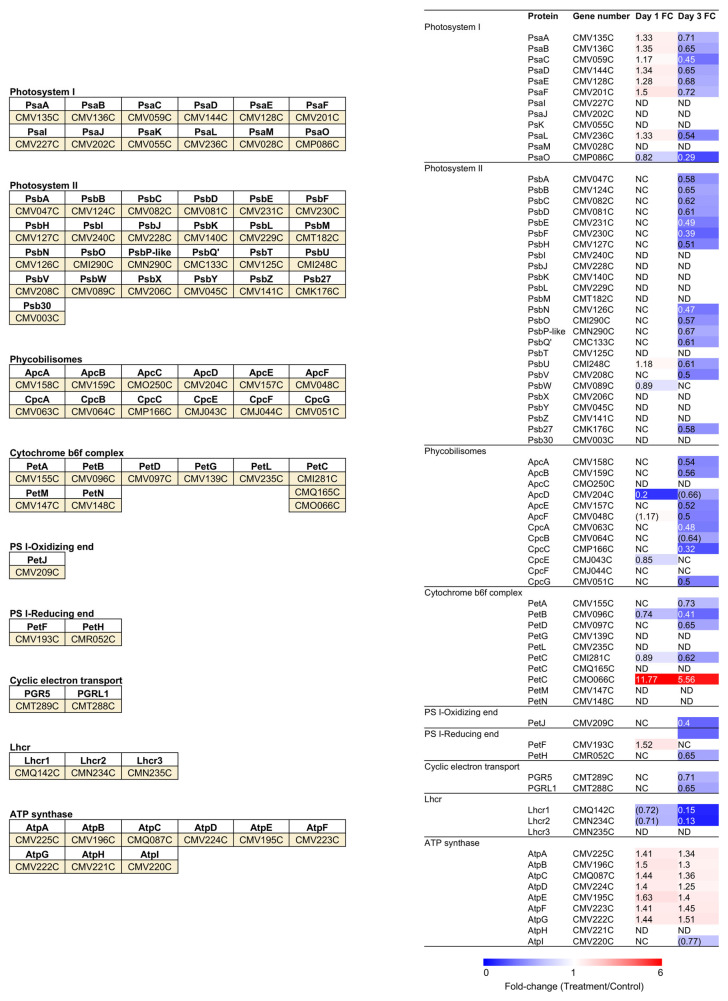
Expression levels of proteins involved in photosynthesis under rapamycin treatment on days 1 and 3 in *C. merolae*. The gene information is based on [103]. The table on the right shows the proteins along with their fold-change (FC) values on days 1 and 3, with colors denoting the degree of fold-change: red denotes an increase in the protein level, blue a decrease. Proteins that show no significant change at the FDR < 0.05 cutoff and those not detected are denoted as NC and ND, respectively. Proteins for which the FC values (in parentheses) were deemed significant after FDR correction (FDR < 0.05) had corresponding raw *p*-values greater than 0.05.

The abundance of photosynthesis-related proteins under rapamycin treatment on days 1 and 3 is shown in Figure 6.

Prolonged exposure to rapamycin led to a general decrease in the levels of photosynthesis-related proteins, with modest changes at day 1 and a more pronounced reduction evident by day 3.

Specifically, reduced abundance was observed for proteins associated with the photosynthetic apparatus, including Photosystem I (PSI), Photosystem II (PSII), cytochrome b6f, phycobilisomes, and the electron transport chain, upon rapamycin treatment in *C. merolae*.

In contrast, the protein content of most, if not all, of the subunits of the F-type ATP synthase was increased. One possible explanation is that these proteins also reflect mitochondrial ATP synthase, as oxidative phosphorylation was upregulated under rapamycin treatment (Table S6). It is likely that ATP production is functionally favored, as cells boost their ATP production as an adaptive compensatory response to the stress conditions generated under rapamycin-inactivated TOR [104]. Additionally, PetC (CMO066C), a Rieske iron–sulfur protein [40], showed markedly increased abundance under rapamycin treatment. In this context, one plausible hypothesis is that TOR signaling indirectly modulates the abundance of PetC (a Rieske iron–sulfur protein) as part of an adaptive remodeling of the photosynthetic electron transport chain, thereby adjusting electron flow and ATP production in response to altered growth and metabolic states.

**Figure 7:**
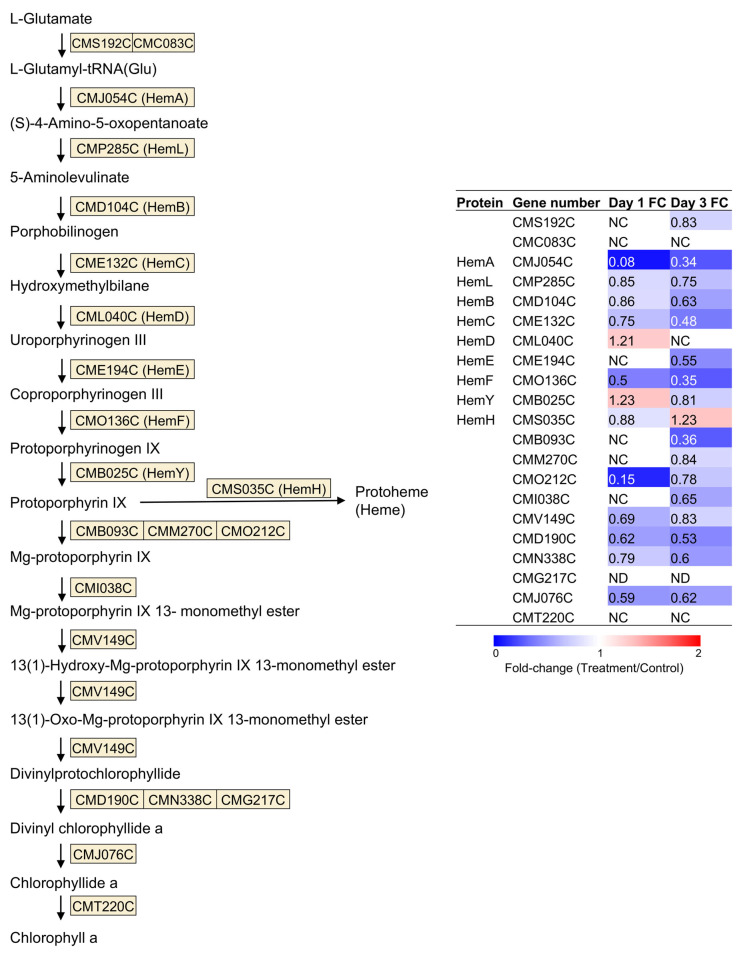
Expression levels of proteins involved in porphyrin metabolism, including chlorophyll biosynthesis, under rapamycin treatment on days 1 and 3 in *C. merolae*. The gene information is based on KEGG Pathway map cme00860 ([96,107,108]). KEGG gene information was obtained via a KEGG mirror site under a commercial-use license. The table on the right shows the proteins along with their fold-change (FC) values on days 1 and 3, with colors denoting the degree of fold-change: red denotes an increase in the protein level, blue a decrease. Proteins that show no significant change at the FDR < 0.05 cutoff and those not detected are denoted as NC and ND, respectively.

Among the proteins whose abundance decreased, a PSI subunit, PsaO (CMP086C), showed a 3.4-fold decrease. In addition, light-harvesting chlorophyll proteins Lhcr1 (CMQ142C) and Lhcr2 (CMN234C) were greatly reduced, with levels decreasing 6.7-fold and 7.7-fold, respectively. Consistent with these findings, Ref. [66] reported significant downregulation of the genes encoding the light-harvesting protein complex proteins such as LHCA1 and LHCA2; notably, red algal Lhcr proteins are homologous to the LHCI proteins of the green lineage. Because both Lhcr proteins and PsaO require chlorophyll for proper functioning [105], their reduced abundance suggests diminished chlorophyll availability under rapamycin treatment. Supporting this interpretation, GO:BP analysis (Figure 2d) showed that porphyrin metabolism, which includes chlorophyll biosynthesis, was significantly reduced under rapamycin treatment. Investigation of individual enzymes involved in chlorophyll biosynthesis further revealed that most, if not all, decreased in abundance (Figure 7), a trend similar to that reported in *Nannochloropsis gaditana* under rapamycin-inactivated TOR [66]. According to Ref. [106], the lack of chlorophyll suppresses the de novo assembly and accumulation of PSI and may also influence the chlorophyll PSII antenna size through post-transcriptional regulation.

Overall, the role of TOR signaling in photosynthesis appears complex and is yet to be fully understood. Previous work on microalgae has demonstrated the role of TOR in photosynthesis, such as in regulating the functional states of both PSII and PSI and maintaining their relative abundance [104], in cyst structure and chlorophyll biosynthesis [66]. In this study, TOR inactivation broadly affected photosynthesis-related proteins, including components of PSII and PSI, photosynthetic pigments, phycobilisomes, and the electron transport chain. These results suggest a general role of TOR kinase in regulating photosynthesis and point to a conserved regulatory role across diverse microalgae.

#### HemA and Mg-ProtoIX Protein Content Is Greatly Reduced Under the TOR Inactivation State

Under TOR inactivation conditions, we observed a general decrease in the abundances of proteins involved in porphyrin metabolism (Figure 7). Among these decreased proteins, the levels of CMJ054C (HemA) and CMO212C were reduced by 12.5-fold and 6.7-fold, respectively, on day 1 (Figure 7). CMO212C catalyzes the conversion of Protoporphyrin IX to Mg-protoporphyrin IX (Mg-ProtoIX). The decline in Mg-ProtoIX has been reported to affect organelle DNA replication (ODR) and nuclear DNA replication (NDR) as Mg-ProtoIX acts as a retrograde signal to coordinate ODR and NDR in *C. merolae* [109]. Reduced porphyrin metabolism due to TOR inactivation may also be linked to changes in processes associated with DNA replication. This possibility is supported by the association of TOR inhibition with reduced abundance of cell-cycle-related proteins observed in this study (Figure 2b,d). CMJ054C (HemA) is glutamyl-tRNA reductase, which catalyzes the first step specific to porphyrin biosynthesis. Ref. [110] reported that Mg-ProtoIX induces the accumulation of HemA and was proposed to function as an organellar signal in regulating HemA expression. Therefore, it is reasonable to assume that the low levels of Mg ProtoIX may contribute to the decrease, or more precisely, to the lack of increase, in the levels of HemA (CMJ054C) in *C. merolae*.

## 4. Conclusions

In this study, we investigated the impact of TOR inactivation on the proteome of the unicellular red alga *C. merolae*. Quantitative proteomic analysis revealed that TOR inhibition induces global metabolic remodeling at the protein level, extending beyond the transcriptional regulation previously reported in microalgae. TOR inactivation broadly suppressed protein synthesis and photosynthetic machinery, indicating reduced growth capacity, and was associated with proteomic changes consistent with altered carbon allocation toward storage-related pathways. In particular, proteins involved in TAG and starch metabolism were preferentially upregulated, highlighting a shift in carbon partitioning under TOR-inhibited conditions. Among these, DGAT and UGP emerged as candidate enzymes that may be leveraged to enhance TAG and starch accumulation, respectively. Importantly, our findings indicate that TOR functions as a central coordinator of interconnected metabolic pathways rather than as a direct regulator of individual biochemical reactions. While TOR inhibition favors the accumulation of biofuel-relevant carbon reserves, it also imposes trade-offs in cellular fitness by suppressing growth-related processes. Our proteomic dataset provides a foundational reference for future studies, combining metabolic flux analysis and genetics-based functional validation approaches to elucidate how TOR signaling balances growth and carbon storage in microalgae.

## Data Availability

The complete proteomics datasets are available from the corresponding author upon reasonable request. The data are not publicly available due to their large size and specialized format.

## Supplementary Materials

The following supporting information can be downloaded at: https://www.mdpi.com/article/10.3390/plants15121790/s1, Figure S1: Principal component analysis (PCA) of protein-group-level intensities; Table S1: Expression levels of proteins associated with fatty acid biosynthesis under rapamycin treatment on days 1 and 3 in *C. merolae*; Table S2: Expression levels of proteins associated with nitrogen assimilation under rapamycin treatment on days 1 and 3 in *C. merolae*; Table S3: Expression levels of proteins associated with ribosome biogenesis under rapamycin treatment on days 1 and 3 in *C. merolae*; Table S4: Expression levels of proteins associated with RNA polymerase under rapamycin treatment on days 1 and 3 in *C. merolae*; Table S5: Expression levels of proteins associated with the ribosome under rapamycin treatment on days 1 and 3 in *C. merolae;* Table S6: Expression levels of proteins associated with oxidative phosphorylation under rapamycin treatment on days 1 and 3 in *C. merolae*.
