# Supplementary material for: Target of Rapamycin Coordinates Metabolic Remodeling at the Protein Level in the Red Alga Cyanidioschyzon merolae
Source: Plants (Basel). 2026 Jun 10;15(12):1790. doi: 10.3390/plants15121790 (PMC13306765; doi:10.3390/plants15121790)
Supplement: Supplementary file 1 [file plants-15-01790-s001.zip › plants-4316072-supplementary.pdf]

Supplementary Material

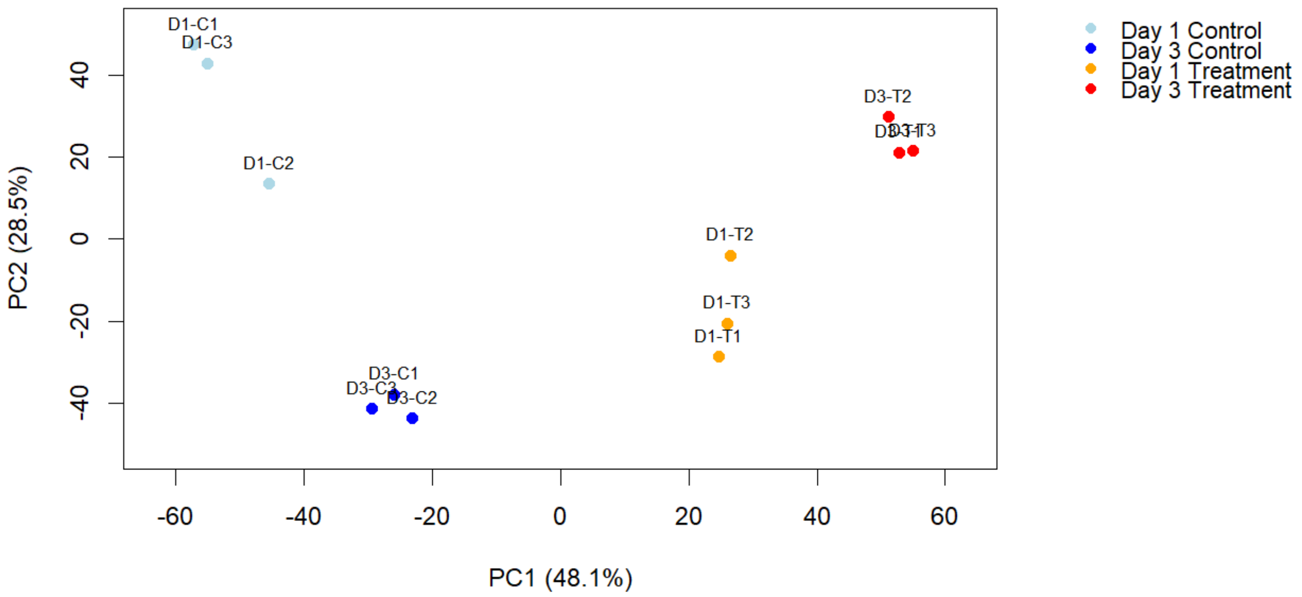

Figure S1. Principal component analysis (PCA) of protein group–level intensities. Each point represents an individual replicate (n = 3 per condition). Samples cluster by experimental group and time point, demonstrating separation among all four conditions and overall reproducibility. Colors denote sample groups as follows: light blue, Day 1 control (D1-C); dark blue, Day 3 control (D3-C); orange, Day 1 treatment (D1-T); and red, Day 3 treatment (D3-T). Replicates are labeled on the plot with abbreviated group names followed by replicate numbers (e.g., D1-C1, D1-C2, D1-C3).

Table S1. Expression levels of proteins associated with fatty acid biosynthesis under rapamycin treatment on days 1 and 3 in *C. merolae*. The gene information is based on [22]. The table shows the proteins along with their fold-change (FC) values on day 1 and day 3, with colors denoting the degree of change: red denotes an increase in the protein level, blue a decrease. Proteins that show no significant change at an FDR < 0.05 cutoff are denoted as NC. Proteins for which the FC values (in parentheses) were deemed significant after FDR correction (FDR < 0.05) but had corresponding raw *p*-values greater than 0.05.

Protein	Gene number	Day 1 FC	Day 3 FC
Biotin carboxylase	CMS299C	0.7	0.58
Malonyl-CoA ACP transacylase	CMT420C	NC	0.57
3-ketoacyl-ACP synthase	CMM286C	0.78	0.55
3-ketoacyl-ACP synthase	CML329C	1.54	1.35
3-ketoacyl-ACP synthase	CMD118C	0.45	0.26
3-ketoacyl-ACP reductase	CMS393C	NC	0.74
3-hydroxyacyl-ACP dehydratase	CMI240C	(0.85)	(0.85)
Enoyl-ACP reductase	CMT381C	NC	0.87

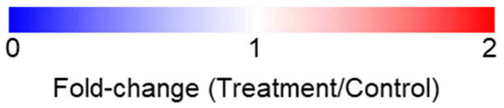

Table S2. Expression levels of proteins associated with nitrogen assimilation under rapamycin treatment on days 1 and 3 in *C. merolae*. The gene information is based on [95]. The table shows the proteins along with their fold-change (FC) values on day 1 and day 3, with colors denoting the degree of change: red denotes an increase in the protein level, blue a decrease. Proteins that show no significant change at an FDR < 0.05 cutoff and those not detected are denoted as NC and ND, respectively. (NRT: Nitrate transporter; NR: Nitrate reductase; NiR: Nitrite reductase; GS: Glutamine synthetase; MYB1: Nitrogen transcription factor; AMT2/1: Ammonium transporter; GOGAT: Glutamate synthase)

Protein	Gene number	Day 1 FC	Day 3 FC
NRT	CMG018C	ND	ND
NR	CMG019C	ND	NC
NiR	CMG021C	16.70	36.72
GS	CMI233C	1.63	1.59
MYB1	CMJ282C	NC	0.16
AMT2	CMK126C	ND	ND
AMT1	CMT526C	1.50	2.72
GOGAT	CMV060C	1.57	1.16

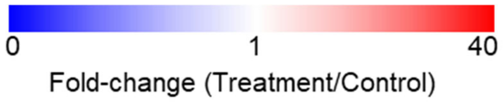

Table S3. Expression levels of proteins associated with ribosome biogenesis under rapamycin treatment on days 1 and 3 in *C. merolae*. The gene information is based on KEGG pathway cme03008 obtained via a KEGG mirror site under a commercial-use license [96]. The table shows the proteins along with their fold-change (FC) values on day 1 and day 3, with colors denoting the degree of change: red denotes an increase in protein level, blue a decrease. Proteins that show no significant change at an FDR < 0.05 cutoff are denoted as NC. Proteins for which the FC values (in parentheses) were deemed significant after FDR correction (FDR < 0.05) but had corresponding raw *p*-values greater than 0.05.

Gene number	Day 1 FC	Day 3 FC
CMB019C	0.44	NC
CMB146C	0.44	1.38
CMC010C	0.57	(0.8)
CMC063C	0.31	0.68
CME044C	0.7	1.27
CMG027C	NC	3.24
CMG096C	(1.45)	(1.35)
CMG105C	0.67	1.38
CMG167C	0.66	1.48
CMG184C	0.4	NC
CMH198C	NC	NC
CMH278C	0.55	NC
CMI018C	0.46	NC
CMI098C	0.87	0.8
CMI163C	NC	(1.15)
CMI184C	(0.8)	(1.24)
CMJ083C	0.36	1.49
CMJ120C	0.71	2.23
CMJ206C	0.43	NC
CML046C	0.72	(1.15)
CMM005C	1.23	1.52
CMM044C	0.79	NC
CMM152C	NC	NC
CMN074C	0.7	0.76
CMN132C	0.81	NC
CMN167C	0.67	2.89
CMO008C	0.82	NC
CMP128C	0.85	1.18
CMP335C	(1.2)	NC
CMQ071C	0.5	NC
CMQ185C	0.8	0.75

CMQ312C	0.5	NC
CMQ316C	0.85	NC
CMQ345C	0.76	NC
CMQ382C	0.62	1.14
CMR019C	0.44	1.24
CMR143C	0.82	0.72
CMR371C	0.43	0.74
CMR447C	NC	1.11
CMS060C	0.37	NC
CMS124C	NC	1.5
CMS199C	0.64	1.29
CMS276C	NC	1.13
CMS302C	0.85	NC
CMT043C	(0.75)	1.84
CMT130C	0.7	1.63
CMT194C	NC	1.48
CMT257C	0.75	0.89
CMT315C	NC	0.89
CMT351C	NC	1.65
CMT444C	0.37	0.89
CMT453C	0.49	NC
CMT605C	0.81	0.78

0 1 4

Fold-change (Treatment/Control)

Table S4. Expression levels of proteins associated with RNA Polymerase under rapamycin treatment on days 1 and 3 in *C. merolae*. The gene information is based on KEGG pathway cme03020 obtained via a KEGG mirror site under a commercial-use license [96]. The table shows the proteins along with their fold-change (FC) values on day 1 and day 3, with colors denoting the degree of change: red denotes an increase in the protein level, blue a decrease. Proteins that show no significant change at FDR < 0.05 cutoff and those not detected are denoted as NC and ND, respectively. Proteins for which the FC values (in parentheses) were deemed significant after FDR correction (FDR < 0.05) but had corresponding raw *p*-values greater than 0.05.

Protein	Gene number	Day 1 FC	Day 3 FC
RNA polymerase III subunit C34	CMB005C	0.49	0.73
DNA-directed RNA polymerase subunit CX	CMC191C	ND	ND
RNA polymerase II subunit G	CMD026C	0.77	NC
DNA-directed RNA polymerase I subunit	CME024C	ND	ND
RNA polymerase III second largest subunit	CME101C	NC	1.63
Probable RNA polymerase II subunit I	CMG169C	NC	NC
RNA polymerase III largest subunit	CMG179C	0.8	0.79
RNA polymerase I, II, III common subunit	CMH017C	ND	ND
RNA polymerase II second largest subunit	CMH117C	0.8	NC
RNA polymerase I largest subunit	CMK037C	NC	1.45
RNA polymerase I, III common subunit	CMK059C	0.73	NC
RNA polymerase III subunit	CMO075C	NC	(0.81)
RNA polymerase II subunit	CMP008C	NC	NC
RNA polymerase II third large subunit	CMP043C	0.83	NC
RNA polymerase I, II, III common subunit	CMQ056C	0.83	NC
RNA polymerase I, III common subunit	CMR197C	0.59	NC
RNA polymerase II largest subunit	CMR224C	0.87	NC
RNA polymerase I, II, III common subunit	CMR232C	0.78	NC
RNA polymerase I second largest subunit	CMS114C	0.76	1.44
RNA polymerase I, II, III common subunit	CMT451C	(0.85)	1.17
RNA polymerase II subunit	CMT583C	0.76	NC
DNA-directed RNA polymerase alpha chain	CMV185C	0.89	0.85
DNA-directed RNA polymerase beta chain	CMV216C	0.81	NC
DNA-directed RNA polymerase beta' chain	CMV217C	0.83	NC

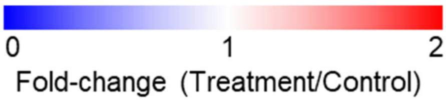

Table S5. Expression levels of proteins associated with the ribosome under rapamycin treatment on days 1 and 3 in *C. merolae*. The gene information is based on KEGG pathway cme03010 obtained via a KEGG mirror site under a commercial-use license [96]. The table shows the proteins along with their fold-change (FC) values on day 1 and day 3, with colors denoting the degree of change: red denotes an increase in protein level, blue a decrease. Proteins that show no significant change at FDR < 0.05 cutoff and those not detected are denoted as NC and ND, respectively. Proteins for which the FC values (in parentheses) were deemed significant after FDR correction (FDR < 0.05) but had corresponding raw *p*-values greater than 0.05.

Gene number	Day 1 FC	Day 3 FC
CMA067C	NC	NC
CMA082C	0.62	0.65
CMA114C	0.59	0.68
CMA122C	0.79	0.82
CMB004C	0.69	0.7
CMB018C	0.57	0.65
CMC044C	0.6	0.65
CMC045C	1.36	1.14
CMC053C	0.69	0.7
CMC090C	1.15	NC
CMC132C	NC	NC
CMC145C	0.74	0.73
CMD103C	NC	NC
CMD140C	0.65	0.66
CME046C	ND	ND
CME054C	0.71	0.72
CME175C	0.47	0.46
CME190C	NC	NC
CMF065C	1.14	NC
CMG057C	NC	0.71
CMG062C	NC	0.83
CMG109C	0.69	0.74
CMG118C	0.82	0.77
CMG157C	0.72	0.72
CMH046C	0.69	0.75
CMH065C	0.72	0.76
CMH066C	1.15	NC
CMH071C	0.7	0.78
CMH218C	0.63	0.7
CMH224C	NC	NC
CMH275C	1.33	1.17
(continued)		
CMN126C	0.62	0.69
CMN148C	0.71	0.69
CMN315C	0.66	0.66
CMO024C	0.7	0.77
CMO062C	ND	ND
CMO287C	0.67	0.77
CMO302C	0.76	0.74
CMO310C	0.69	0.71
CMP006C	0.46	0.48
CMP007C	0.64	0.7
CMP012C	0.44	0.5
CMP175C	0.75	0.73
CMP179C	0.77	0.74
CMP301C	NC	NC
CMQ051C	0.75	0.75
CMQ078C	0.73	0.75
CMQ118C	(0.69)	(0.72)
CMQ138C	(1.33)	NC
CMQ175C	NC	NC
CMQ292C	0.64	0.75
CMQ293C	0.69	0.7
CMQ421C	NC	NC
CMQ445C	0.78	0.78
CMQ463C	0.72	0.76
CMR122C	0.68	0.73
CMR148C	0.69	0.73
CMR150C	0.76	0.67
CMR179C	1.22	NC
CMR201C	1.18	(0.85)
CMR287C	0.73	0.71

CMH286C	0.6	0.73
CMI055C	NC	NC
CMI059C	0.59	0.79
CMI067C	0.67	0.78
CMI140C	ND	ND
CMI202C	0.71	0.77
CMI205C	0.69	0.72
CMI283C	0.67	0.68
CMJ057C	0.56	0.52
CMJ109C	0.77	0.73
CMJ110C	0.44	0.33
CMJ125C	0.62	0.68
CMJ170C	0.74	0.71
CMJ183C	ND	ND
CMJ235C	NC	NC
CMJ238C	NC	NC
CMJ285C	0.72	0.68
CMJ289C	0.67	(0.79)
CMK118C	0.7	0.7
CMK273C	0.76	0.75
CMK274C	NC	NC
CMK296C	ND	ND
CML106C	0.59	0.75
CML196C	0.69	(0.77)
CML304C	0.74	0.85
CML305C	0.75	0.67
CML317C	0.73	0.75
CMM040C	0.72	0.7
CMM190C	0.62	0.85
CMM240C	0.72	0.7
CMM253C	0.62	0.65
CMN112C	NC	0.83
CMN125C	0.75	0.75

(continued)

CMS040C	NC	0.87
CMS047C	1.17	1.18
CMS080C	0.75	0.72
CMS081C	NC	0.83
CMS139C	NC	NC
CMS189C	0.71	0.71
CMS262C	0.68	0.66
CMS284C	0.76	0.77
CMS417C	NC	NC
CMS499C	NC	NC
CMT030C	0.66	0.69
CMT154C	0.61	0.72
CMT159C	0.68	0.79
CMT160C	0.42	1.13
CMT197C	0.77	0.69
CMT330C	NC	NC
CMT378C	NC	NC
CMT402C	0.62	0.67
CMT410C	0.81	0.75
CMT485C	NC	0.89
CMT508C	NC	NC
CMT544C	ND	ND
CMT627C	0.7	0.68
CMV011C	0.59	0.69
CMV035C	0.63	0.71
CMV046C	ND	ND
CMV085C	ND	ND
CMV103C	0.66	0.74
CMV137C	0.71	0.77
CMV168C	0.74	0.67
CMV174C	0.63	(0.82)
CMV182C	0.63	0.67
CMV187C	0.65	0.8
CMV189C	0.7	0.67
CMV190C	0.71	0.8

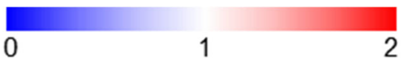

Fold-change (Treatment/Control)

Table S6. Expression levels of proteins associated with oxidative phosphorylation under rapamycin treatment on days 1 and 3 in *C. merolae*. The gene information is based on KEGG pathway cme00190 obtained via a KEGG mirror site under a commercial-use license [96]. The table shows the proteins along with their fold-change (FC) values on day 1 and day 3, with colors denoting the degree of change: red denotes an increase in the protein level, blue a decrease. Proteins that show no significant change at FDR < 0.05 cutoff and those not detected are denoted as NC and ND, respectively. Proteins for which the FC values (in parentheses) were deemed significant after FDR correction (FDR < 0.05) but had corresponding raw *p*-values greater than 0.05.

Gene number	Day 1 FC	Day 3 FC
CMA090C	1.32	1.19
CMC099C	NC	0.84
CMC151C	0.78	NC
CMD007C	NC	1.17
CMD048C	1.25	NC
CMD095C	1.27	1.23
CME198C	NC	NC
CMF056C	1.66	1.71
CMG004C	ND	ND
CMG064C	1.66	1.72
CMG215C	NC	0.7
CMH197C	1.19	1.17
CMH200C	NC	1.19
CMI095C	1.16	0.9
CMI118C	(1.19)	NC
CMI200C	1.25	(1.27)
CMI262C	1.15	NC
CMJ036C	NC	NC
CMJ185C	1.21	NC
CMJ211C	1.26	NC
CMJ212C	NC	0.48
CMJ245C	NC	0.8
CMK031C	1.25	NC
CMK135C	ND	ND
CMK178C	1.12	1.19
CML029C	NC	(1.14)
CML098C	NC	NC
CMM026C	1.83	2.3
CMM030C	(1.24)	NC
CMM034C	1.22	NC
CMM267C	1.21	NC

CMN078C	NC	0.8
CMN127C	NC	NC
CMN203C	1.52	1.36
CMN223C	NC	NC
CMN320C	1.18	(0.89)
CMO080C	1.2	NC
CMO274C	NC	NC
CMP123C	NC	1.34
CMP152C	1.3	0.85
CMQ010C	NC	NC
CMQ087C	1.44	1.36
CMQ200C	1.33	NC
CMQ237C	NC	1.74
CMQ247C	1.54	2.34
CMQ323C	NC	NC
CMQ386C	NC	1.11
CMQ432C	1.21	NC
CMR178C	1.24	0.69
CMR188C	1.13	NC
CMR255C	NC	0.68
CMR289C	NC	0.88
CMR404C	(1.24)	NC
CMS223C	1.29	NC
CMS332C	ND	ND
CMS342C	NC	1.34
CMS372C	NC	NC
CMS431C	1.54	1.46
CMT119C	NC	1.31
CMT198C	1.26	1.15
CMT232C	NC	ND
CMT434C	1.16	1.15
CMT582C	1.56	1.44
CMV195C	1.63	1.4
CMV196C	1.5	1.3
CMV220C	NC	(0.77)
CMV221C	ND	ND
CMV225C	1.41	1.34

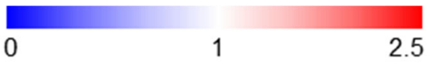

Fold-change (Treatment/Control)
